# Telephone-assisted cardiopulmonary resuscitation in a simulated environment: A pilot exploratory study evaluating first responder adherence and dispatcher performance

**DOI:** 10.1016/j.resplu.2025.101149

**Published:** 2025-11-01

**Authors:** Jordi Caplliure-Llopis, Laura Bonet, Gabriele Kersulyte, Josep Primo-Paris, Ana Micó-Máñez, Carmen Escriche-López, Jose Enrique de la Rubia Ortí

**Affiliations:** a Department of Nursing, Faculty of Medicine and Health Sciences, Catholic University of Valencia San Vicente Mártir, Quevedo, 2, 46003 Valencia, Spain; b Emergency Medical Services of the Valencian Community (SESCV), Av. Campanar 21, 46009 Valencia, Spain; c University Research Institute of Musculoskeletal Disorders, Catholic University of Valencia San Vicente Mártir, Quevedo, 2, 46003 Valencia, Spain; d Department of Basic Biomedical Sciences, Catholic University of Valencia San Vicente Mártir, Quevedo, 2, 46003 Valencia, Spain

**Keywords:** Out-of-hospital cardiac arrest, Telephone-assisted CPR, Dispatcher performance, First responder adherence, Emergency medical services

## Abstract

**Background:**

Out-of-hospital cardiac arrest (OHCA) remains a major public health challenge, and telephone-assisted cardiopulmonary resuscitation (T-CPR) is a key strategy to improve survival. However, real-time analyses of dispatcher–rescuer interaction are scarce.

**Methods:**

A pilot study was conducted in the Valencian Community (Spain) using simulated OHCA scenarios to evaluate first responder adherence to T-CPR instructions and dispatcher performance. This exploratory study included six untrained lay callers and six dispatchers (five nurses, one physician) who participated in simulated emergency calls routed through the regional emergency coordination center (CICU). All calls were audiovisually recorded and analysed using a checklist derived from ERC 2021/AHA 2020 guidelines. Technical CPR parameters were collected from a manikin with QCPR technology.

**Results:**

Time to cardiac arrest recognition varied widely (50 s–3:48 min), with overall delays exceeding 5 min in two cases. Basic instructions (supine position, hand placement) were consistently communicated, but only 50 % of dispatchers emphasized correct compression depth. Interruptions in CPR occurred in four of six cases. Lay responders reliably followed simple instructions but struggled with technical aspects: only 33.3 % achieved adequate compression depth and rate. Significant correlations were observed between compression depth, rate, and rescuer age (ρ = –0.868; p = 0.025). AED use was systematically omitted.

**Conclusions:**

This pilot study highlights variability in dispatcher recognition times and inconsistencies in communication of critical CPR parameters. First responders adhered better to simple than technical instructions. Findings underscore the need for structured, standardized protocols and enhanced dispatcher training to optimize T-CPR effectiveness.

## Introduction

Out-of-hospital cardiac arrest (OHCA) is one of the leading causes of mortality in developed countries, with an estimated incidence of 38–59.9 cases per 100,000 inhabitants per year and a hospital discharge survival rate below 10 %.^1^, ^2^ Cardiopulmonary resuscitation (CPR) initiated by bystanders within the first 4 min can triple survival and reduce neurological damage^3^, ^4^; however, manual chest compressions achieve only 20–30 % of normal cardiac output, highlighting the need to optimize their quality.^5^, ^6^

Essential lifesaving basic life support (BLS) manoeuvres, such as chest compressions, rescue breaths, and the use of an automated external defibrillator (AED), can be performed by any bystander, whether layperson or healthcare professional. Accordingly, several European countries (including Norway, Denmark, France, the United Kingdom, and Spain) supported by the International Liaison Committee on Resuscitation (ILCOR) “Kids Save Lives” initiative, have developed and incorporated CPR training programs into school curricula.^7^, ^8^, ^9^ In Spain, implementation remains partial and heterogeneous, with limited general population knowledge.

Data from the Out-of-Hospital Spanish Cardiac Arrest Registry (OHSCAR) for 2017–2018 show that 77.8 % of OHCAs were witnessed, and in 66.1 % of these cases, the witness was a layperson.^9^ However, 47.1 % of patients did not receive BLS; only 52.9 % received any early intervention, and of these, 51.7 % were performed by laypersons. Currently, bystander CPR occurs in 39.3 % of cases, and AED use by laypeople is only 4 %.^8^ Although most cardiac arrests are witnessed, only 39–52 % of patients receive bystander CPR in Spain, which is below the European average.^8^, ^9^

In comparison, the 2017 European Registry of Cardiac Arrest TWO (EuReCa TWO), which included data from 29 countries, estimates an average European rate of bystander CPR by laypersons of 58 %, with national variations between 13 % and 82 %.^10^ In countries such as the Netherlands and Sweden, rates exceed 59 %, whereas some Spanish regions, such as Andalusia, report rates as low as 12 %.^11^

A key strategy to improve survival in OHCA is telephone-assisted CPR (T-CPR), in which emergency dispatch centres help the first responder recognize cardiac arrest and guide the initiation of CPR until the arrival of advanced life support (ALS) units.^12^, ^13^, ^14^ While T-CPR is a recommended strategy, its implementation varies across emergency systems. In countries such as the United States or Japan, T-CPR is typically led by physicians or paramedics. In the Valencian Community (Spain), it is performed interchangeably by nurses or physicians from the Emergency Coordination and Information Centre (CICU), allowing exploration of the nursing role in T-CPR, which remains under-studied.

Although the critical role of T-CPR in identifying signs of cardiac arrest and managing communication is recognized,^14^ barriers such as witness refusal, logistical difficulties, and lack of standardized instructions persist.^15^ Furthermore, no studies have analysed the interaction between dispatchers and first responders at the time of T-CPR limiting the identification of potential improvements. Therefore, it was hypothesized that evaluating the adherence of first responder to the instructions given by dispatcher could provide to the CICU assistants a clearer knowledge about of the quality of the T-CPR manoeuvres delivered by non-professional first responders. This evaluation could also help to develop an operational checklist to optimize the instructions of dispatcher that in some cases may be necessary.

This pilot study, conducted in a simulated environment, aims to assess first responder adherence to T-CPR instructions and the quality of information given by dispatchers of the Valencian Community CICU. A secondary objective was to propose a checklist to improve dispatcher performance, with potential applicability to other emergency systems. As an exploratory study, this work also tried to assess the feasibility of using simulated OHCA scenarios, audiovisual recording, and QCPR technology to evaluate dispatcher performance and first responder adherence.

## Methods

### Study design

A pilot observational study was conducted using simulated OHCA stage designed to evaluate first responder adherence to dispatcher instructions and the quality of these T-CPR instructions.

The study design involved two groups: untrained callers (first responders) and CICU dispatchers (5 nurses and 1 physician).

Although formal feasibility metrics such as recruitment rate or technical reliability were not quantified, the study allowed preliminary verification of the methodological approach and data collection tools.

### Stage and participants

The study included a small sample (n = 6 callers and 6 dispatchers) to assess protocol feasibility and generate hypotheses for future research.^16^

Simulations were conducted between March and April 2025 in six households, located on average 5 km from the ALS unit involved, to reproduce realistic conditions without interfering with actual emergency operations. First responders were volunteers from the general population (3 men and 3 women), selected by convenience sampling, aged 21–77 years, with no prior healthcare training or BLS experience. All participants signed informed consent. Authorization was obtained from the Valencian Emergency Health Services (SESCV) to include simulated calls within the usual 112 workflow, avoiding activation of other agencies.

Dispatchers were not specifically selected; those on duty during the simulation days participated, effectively creating a convenience sample. All participants were unaware of the simulated nature of the calls and provided T-CPR following usual practice. Each caller placed a 112 call from their home, which, upon detection of cardiac arrest, was routed to the CICU.

### Procedure

This study focuses on the Valencian Community (Spain), where emergency calls are centralized via 112 (911 in the U.S.) and medical calls are routed to the CICU under SESCV.

In each simulation, the caller acted as if the manikin (Laerdal Resusci Anne® with QCPR technology) were in cardiac arrest, effectively becoming the first responder. Calls were made in hands-free mode from a mobile phone and audiovisual recording for later analysis. The manikin allowed measurement of technical parameters such as compression rate, depth, and recoil.

Dispatchers guided T-CPR without a structured protocol based mainly on clinical knowledge but following most of the ERC 2021 guideline issues. The dispatchers were blinded to the simulated design of the study. Interventions included confirming cardiac arrest, instructing continuous chest compressions, and activating ALS resources. The ALS team then attended the household to simulate the outcome (asystole or continuation of compressions), preserving study confidentiality. After each simulation, first responders completed a subjective questionnaire, and technical manikin data were extracted for analysis.

Recordings were independently evaluated by each expert and later discussed in a consensus session to resolve discrepancies. The checklist assessed: time to cardiac arrest recognition, time to first compression, compression quality according to ERC 2021 guidelines, adherence to instructions (position, hand placement, chest location), among other parameters. Hand position was visually assessed from video recordings by two independent reviewers using the predefined checklist criteria based on ERC 2021 guidelines.

### Evaluation instrument

The evaluation instrument was developed through a structured, two-phase process. First, an exhaustive review of international CPR guidelines (ERC 2021 and AHA 2020) identified critical parameters for high-quality CPR. A preliminary checklist was then created and refined through consensus with two out-of-hospital emergency professionals (one physician and one nurse with over 10 years of experience), using a modified Delphi method to validate item relevance. Simultaneously, recordings were analysed inductively, refining the instrument to observed practice (Appendix A). This combination of deductive and inductive approaches ensured inclusion of both technical and communicative elements relevant to T-CPR.

### Data analysis

Data were analysed using IBM SPSS v26 (IBM Corp., Armonk, NY, USA). Due to the small sample size, descriptive statistics (median, interquartile range) and non-parametric tests were used. Normality was assessed with the Kolmogorov–Smirnov test; as normality was not assumed (p < 0.05 for all variables), Spearman correlations were applied to assess relationships between quantitative variables (e.g., age vs. compression depth). Mann–Whitney U tests compared groups (e.g., cases with vs. without depth instruction). Qualitative variables (e.g., gender) were analysed with chi-square tests. Cross-tabulations explored adherence and CPR quality. Multivariate analyses were not performed, given the pilot nature of the study.

### Ethical considerations

The study was approved by SESCV management and the Ethics Committee of the Catholic University of Valencia (code UCV/2024-2025/033). All participants signed informed consent, and anonymity and data confidentiality were ensured in accordance with the Declaration of Helsinki, Spanish Biomedical Research Law 14/2007, and GDPR (EU 2016/679). ALS units involved were informed and authorized participation.

## Results

All simulations were successfully completed without major technical incidents, supporting the practical feasibility of the study design.

### CICU performance analysis

The time to cardiac arrest recognition by the CICU dispatcher varied considerably, ranging from 50 s to 3 min and 48 s (Table 1). Cases 5 and 6 showed critical delays. This variability could influence potential survival outcomes.

**Table 1:** Individual response times during T-CPR.

**Case**	**Time to recognize CA (112)**	**Time for CICU to start CPR**	**Dispatcher profession**	**Total time from call start to CPR**
1	0:50	0:40	Nurse	1:30
2	0:59	0:45	Nurse	1:44
3	0:52	0:56	Nurse	1:48
4	2:19	1:30	Nurse	3:49
5	3:48	1:32	Physician	5:20
6	1:26	4:19	Nurse	5:25

### Quality of T-CPR instructions by CICU dispatchers

All dispatchers correctly communicated basic instructions (Table 2). However, appropriate compression depth was indicated by only half of the nurses, compared with all physicians. Omitting this instruction may compromise the hemodynamic efficacy of CPR. Interruptions in CPR were reported in 4 of the 6 cases, with no differences by professional role.

**Table 2:** High-quality chest compression instructions during T-CPR.

**Observed Item**	**Nurses (n = 5)**	**Physician (n = 1)**
Kneel beside patient	3 (60 %)	1 (100 %)
Place patient supine	5 (100 %)	1 (100 %)
Hand placement in centre of chest	5 (100 %)	1 (100 %)
Keep arms straight and hands interlocked	4 (80 %)	1 (100 %)
Place patient on firm surface	4 (80 %)	1 (100 %)
Indicate compression rate (100–120/min)	4 (80 %)	1 (100 %)
Indicate correct compression depth (5–6 cm)	2 (40 %)	1 (100 %)
CPR interrupted at any time	3 (60 %)	1 (100 %)

### First responder analysis

No significant differences were found among first responders in weight, height, or BMI, although age differences were significant: the male group had a mean age of 21.67 years versus 59.67 years for the female group (p < 0.05).

### Concordance between CICU instructions and actions performed

First responders consistently followed basic instructions (Table 3). They also performed actions such as activating hands-free mode or placing the patient on the floor, even if these were not systematically instructed.

**Table 3:** CICU instructions and first responder adherence.

**Instruction/Action**	**CICU (%)**	**First Responder**
Place phone on hands-free/action performed	50 %	83.3 %
Place patient on floor/action performed	83.3 %	100 %
Place patient supine/action performed	100 %	100 %
Kneel beside patient/action performed	66.7 %	50 %
Interlock hands & place on chest centre/action	83.3 %	50 %
Keep arms straight/action performed	100 %	100 %
Compress strongly (5–6 cm)/action performed	50 %	33.3 %
Follow 100–120 compressions/min/action performed	83.3 %	33.3 %
Maintain call until ALS arrival	100 %	100 %

**Fig. 1:**
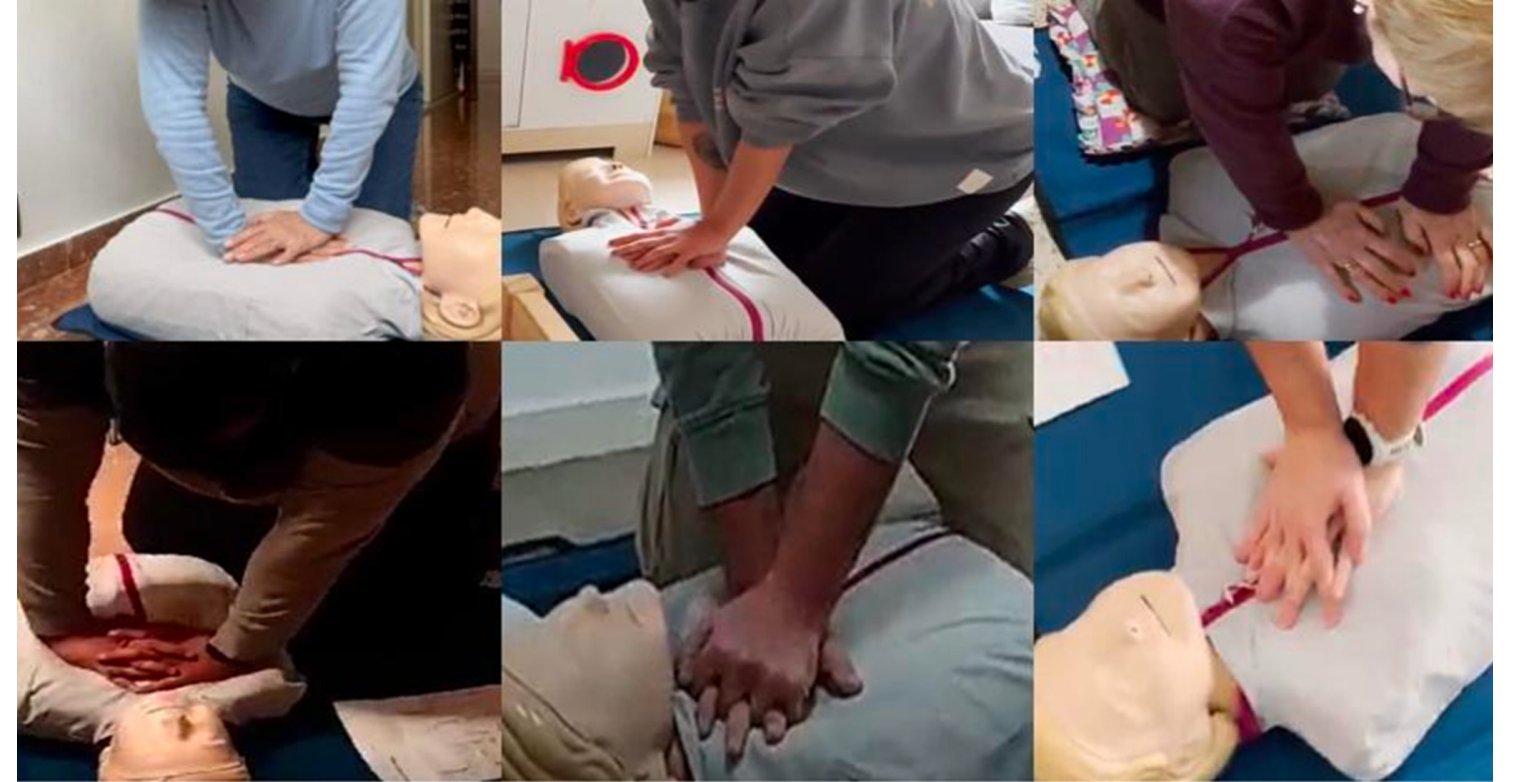
Hand positioning during CPR by first responders.

In contrast, more complex tasks showed lower adherence: only 33.3 % of participants maintained the correct compression rate, and only 33.3 % achieved the recommended depth, despite depth being instructed three times. Correct hand placement was executed in only 50 % of cases (Fig. 1).

### Correlations in technical performance

Significant correlations were found among several technical variables (Table 4). Mean compression depth showed a very strong correlation with maintained adequate depth, suggesting more effective compressions (r = 0.948; p = 0.004), and correct rate correlated with adequate depth (r = 0.919; p = 0.010). Conversely, an almost perfect negative correlation was observed between correct depth and full chest recoil (r = −0.988; p < 0.001), suggesting possible interference between these parameters.

**Table 4:** Correlations between technical CPR quality variables and individual characteristics. Source: authors’ own data.

**Correlated Variables**	**Coefficient (r/ρ)**	**Significance (p)**	**Interpretation**
Mean depth vs. % time with correct depth	0.948	0.004**	Very strong, significant
% correct rate vs. % correct depth	0.919	0.010*	Very strong, significant
% correct depth vs. % full chest recoil	–0.988	<0.001**	Almost perfect negative, significant
Mean depth vs. age	–0.868	0.025*	Strong negative, significant
Mean depth vs. weight	0.691	0.128	Moderate, not significant

* p < 0.05.

** p < 0.01.

Additionally, rescuer age was negatively associated with mean depth (ρ = −0.868; p = 0.025), while weight showed a positive, non-significant correlation (ρ = 0.691; p = 0.128).

## Discussion

This study provides an innovative approach to analysing T-CPR in a simulated environment, representing the first investigation to explore in real time the interaction between T-CPR dispatchers and first responders. Although the sample size is limited, such studies should be evaluated not for statistical power but for their ability to assess feasibility, logistics, and procedural understanding.^17^ These findings allow evaluation of protocol feasibility and generation of hypotheses for future research.

Significant variability was observed in cardiac arrest recognition times and initiation of T-CPR, although overall latencies were within ranges reported in the literature.^18^, ^19^, ^20^ However, delays exceeding 5 min are concerning, as each minute without CPR reduces survival probability by 7–10 % in OHCA.^21^, ^22^ Variability may result from differences in dispatcher experience or clarity of witness communication, highlighting the need for standardized protocols.

Although stage was designed to facilitate cardiac arrest identification, witness communication challenges (anxiety, non-technical language) complicated interpretation. Combined with heterogeneous dispatcher experience, this underscores the importance of standardized protocols and specific communication training to improve early recognition in the Valencian model.

Basic instructions were consistently delivered, but critical aspects for hemodynamic efficacy (such as compression depth and rate) were frequently omitted. This pattern aligns with previous studies,^18^, ^23^ which identified gaps in the communication of technical parameters, without significant differences between physicians and nurses. This suggests that variability is related not to professional competence but to the absence of protocols prioritizing technical quality in T-CPR. Literature confirms that after T-CPR guideline implementation, no differences were observed by professional role,^24^ and that clear protocols improve compliance and quality.^12^, ^24^

Analysis of performed CPR revealed discordance between dispatcher instructions and lay responder actions. Simple instructions were followed faithfully, while more technical manoeuvres showed significantly lower adherence. This highlights the need for sequential, simple, and tailored instructions according to the responder's level. This pattern is consistent with previous reports,^25^, ^26^ emphasizing the difficulty of achieving technically effective CPR in untrained populations, likely influenced by factors such as limited anatomical understanding, ambiguity, fatigue, or emotional insecurity.^25^, ^26^, ^27^, ^28^

In this context, video calls may represent a promising tool to enhance adherence and T-CPR quality by providing direct visual supervision and rapid real-time evaluation.^29^, ^30^, ^31^

A critical finding was the systematic omission of AED retrieval, despite its importance in shockable rhythms.^32^ Considering that 25–30 % of cardiac arrests are due to shockable rhythms (VF/pulseless VT), this represents a clear opportunity for protocol and dispatcher training improvement. Additionally, some first responders interrupted CPR due to uncertainty or insecurity, reinforcing literature recommendations for clear, motivating, and continuous guidance throughout the call.^33^

These findings highlight the importance of providing structured, clear, and positively reinforced telephone instructions, and support the need to strengthen training for both dispatchers and the general population to improve T-CPR efficacy. The effectiveness of T-CPR depends not only on technical content but also on communication quality.^33^

Although the sample size limits result extrapolation, consistent trends with previous studies were observed. Rescuer age negatively correlated with compression depth, reflecting lower performance among older individuals, as reported in the literature.^34^, ^35^ Weight showed a moderate positive correlation, consistent with Cornara et al.^36^ Furthermore, participants achieving greater depth tended also to maintain an adequate and consistent rate, suggesting interrelation among technical components.

Finally, participants who achieved good depth also tended to maintain a constant rate, suggesting that technical CPR elements are interrelated and mutually reinforcing. However, greater depth was negatively associated with complete chest recoil, which could compromise hemodynamic efficacy by hindering cardiac filling. Overall, findings indicate that both individual characteristics and interaction among technical parameters influence CPR quality, reinforcing the need to optimize instructions, protocols, and training strategies for lay responders.

### Limitations

Despite the study's innovation and realism, limitations must be considered. The small sample size, although justified for protocol feasibility assessment, limits statistical power and generalizability. However, this is appropriate given the exploratory pilot design. Additionally, hand position during chest compressions was not objectively quantified, which may limit the precision of CPR quality assessment.

Simulation use cannot fully reproduce the stress or emotional and operational context of real OHCA. Participant awareness of study involvement may have influenced behaviour (Hawthorne effect), and the high educational level of volunteers’ limits applicability to the general population.

Factors such as language barriers, cognitive deficits, or emotional variables were not considered, but could substantially influence T-CPR quality. These limitations suggest avenues for future studies aimed at optimizing telephone CPR implementation and effectiveness in diverse contexts.

### Future research directions

This pioneering study opens several avenues for future research, including expanding sample size and settings, including control groups, and using full-body, weighted manikins for more realistic practical assessment. Developing structured protocols and enhancing dispatcher communication training is essential to study stress impact on first responders and analyse real-time communicative interaction during T-CPR. These actions will improve T-CPR quality and effectiveness, better preparing operators and the general population.

## Conclusions

This pilot study provides preliminary yet insightful evidence on real-world T-CPR dynamics between dispatchers and first responders. Variability was identified in cardiac arrest detection and technical quality of guided CPR. These results underscore the need for structured, comprehensible, and motivating instructions, as well as targeted training strategies for laypeople. As a practical tool, a checklist is proposed to guide emergency call operators during T-CPR.

## CRediT authorship contribution statement

**Jordi Caplliure-Llopis:** Conceptualization, Data curation, Investigation, Methodology, Supervision, Writing – original draft, Writing – review & editing. **Laura Bonet:** Conceptualization, Writing – original draft. **Gabriele Kersulyte:** Investigation, Methodology. **Josep Primo-Paris:** Data curation, Formal analysis, Software. **Ana Micó-Máñez:** Data curation, Project administration, Validation. **Carmen Escriche-López:** Conceptualization, Investigation, Supervision. **Jose Enrique de la Rubia Ortí:** Investigation, Validation, Visualization, Writing – review & editing.

## Declaration of competing interest

The authors declare that they have no known competing financial interests or personal relationships that could have appeared to influence the work reported in this paper.

## Appendix A T-CPR Checklist

1. Check if the person responds.
2. Open airway (one hand on forehead, the other on the chin, head slightly tilted back to help breathing).
3. Confirm whether the caller is alone or with someone.
  o If accompanied, instruct the companion to locate an AED.
  o Notify the nearest health center.
  o Contact local police and inform them to bring an AED.
4. Activate basic and/or advanced mobile resources (BLS and/or ALS).
5. Instruct to put the phone on speaker mode.
6. Place the victim on the floor or a firm, flat surface in a supine position.
7. Expose the chest (cut/remove clothing if necessary).
8. Kneel beside the victim’s chest.
9. Instruct hand placement: palm of one hand on the back of the other, interlock fingers, position in the center of the chest between the nipples, arms straight, shoulders above chest.
  o Use clear, concise, and respectful language.
10. Start timer or record the exact time of first compression.
11. Begin compressions at 100–120/min.
  • Emphasize depth of 5–6 cm.
  • Ensure full chest recoil between compressions.
  • Avoid unnecessary pauses.
12. Instruct to count compressions aloud (to monitor rhythm).
13. Verify that the responder understands instructions.
14. Periodic reassessment (first at 2 min):
  • Breathing?
  • Movements?
15. Ask about terminal illness or other conditions (diabetes, prior chest pain, epilepsy, stroke/TIA, intoxications).
16. Ask if death was expected.
17. Provide verbal encouragement: “You are doing very well.”
18. Give instructions for switching rescuers, if applicable:
  a. Ask if fatigued.
  b. Check rhythm and depth.
19. Collect victim information if possible: SIP, ID, full name.
20. Ask if others are waiting outside for emergency services (police, ambulance).^21^, ^22^, ^23^, ^24^, ^25^, ^26^, ^27^, ^28^, ^29^, ^30^ Repeat periodic reassessments every 2 min, reinforce verbal encouragement, and provide instructions for switching rescuers until ALS arrival.
